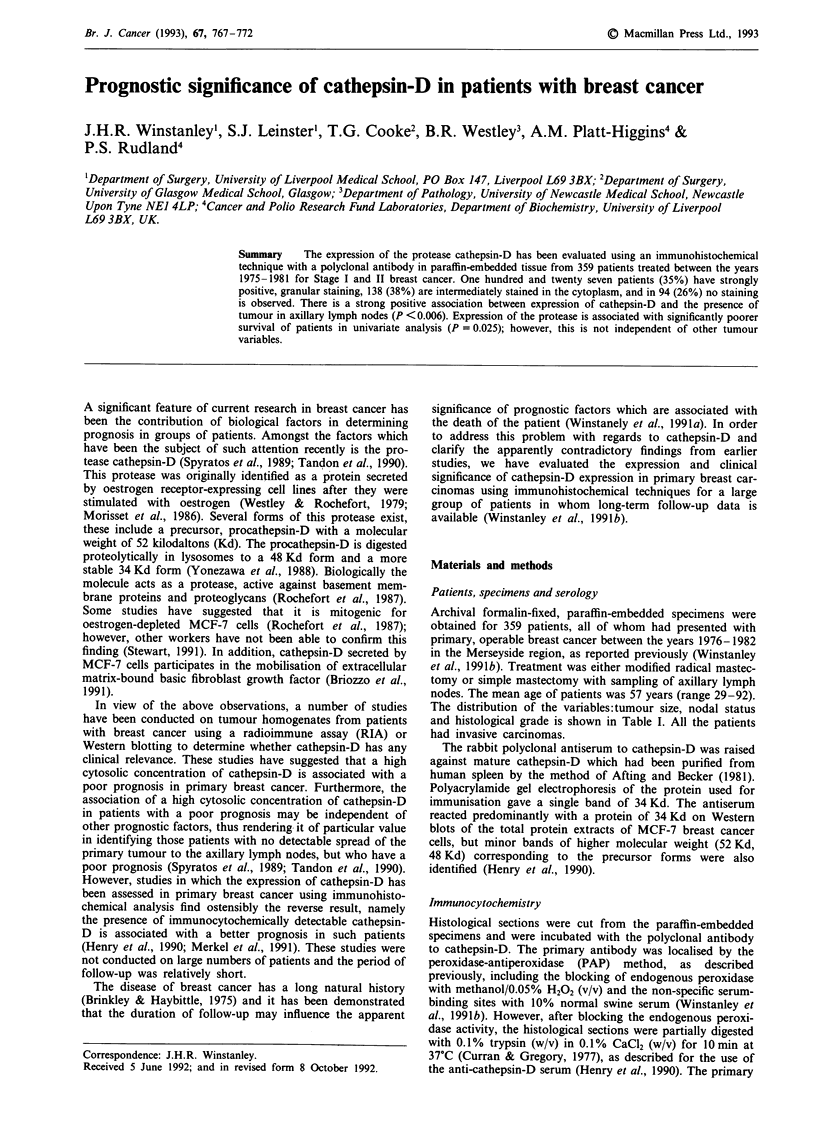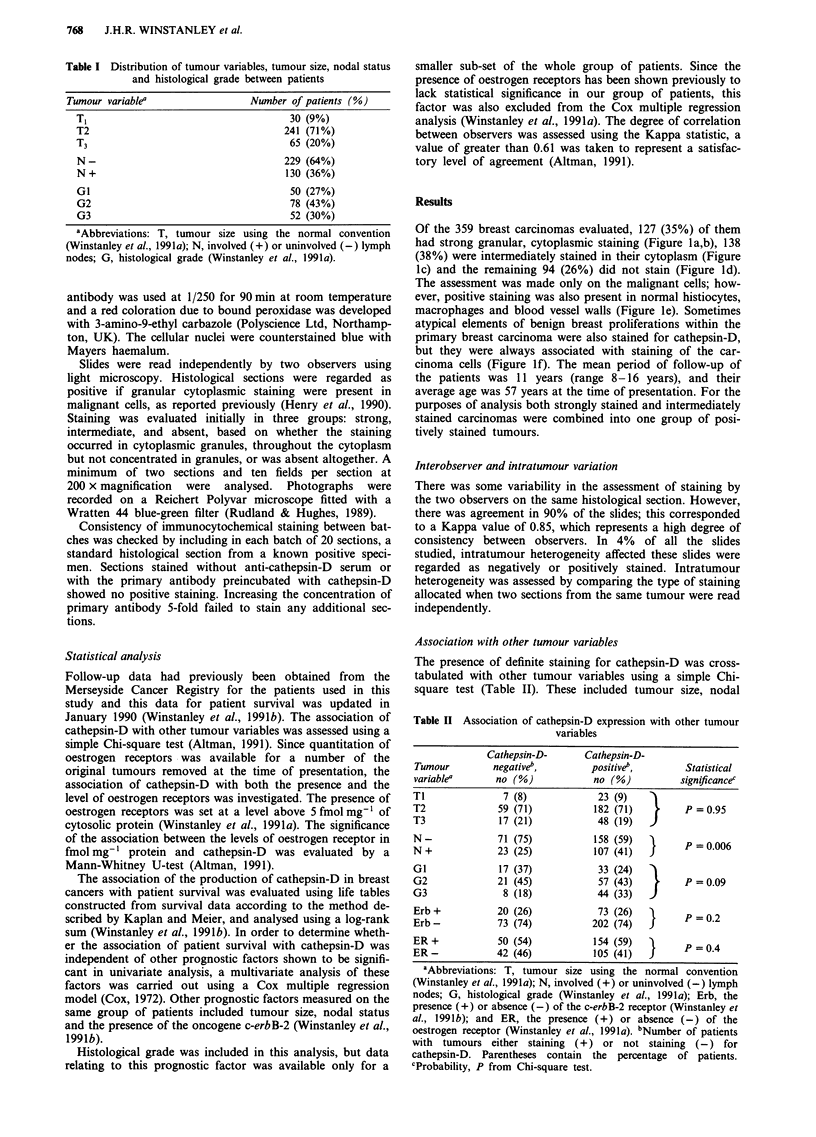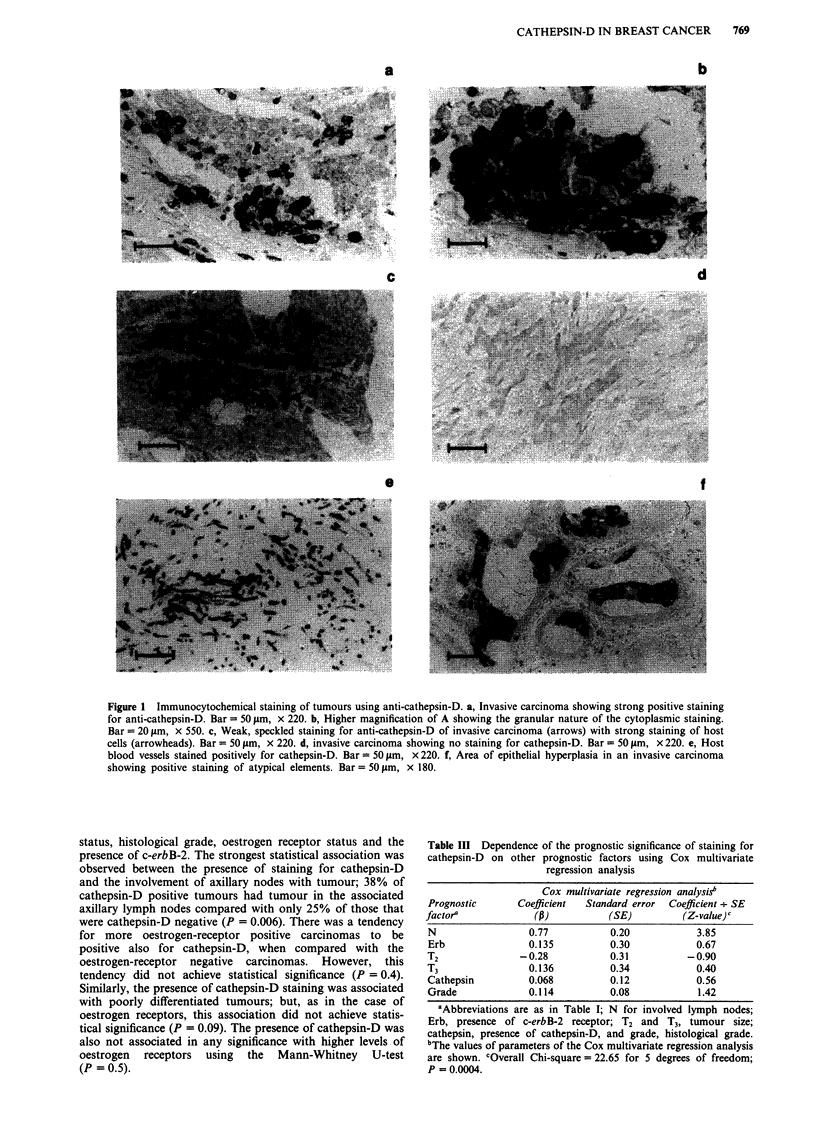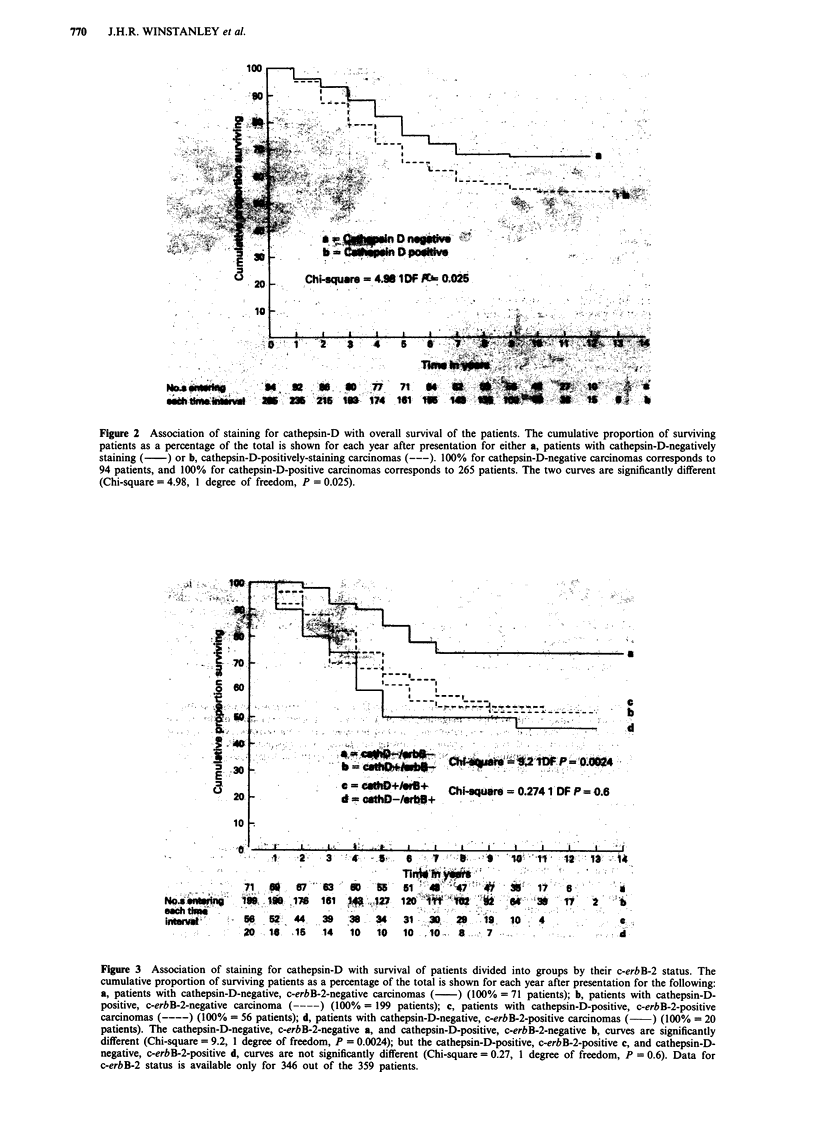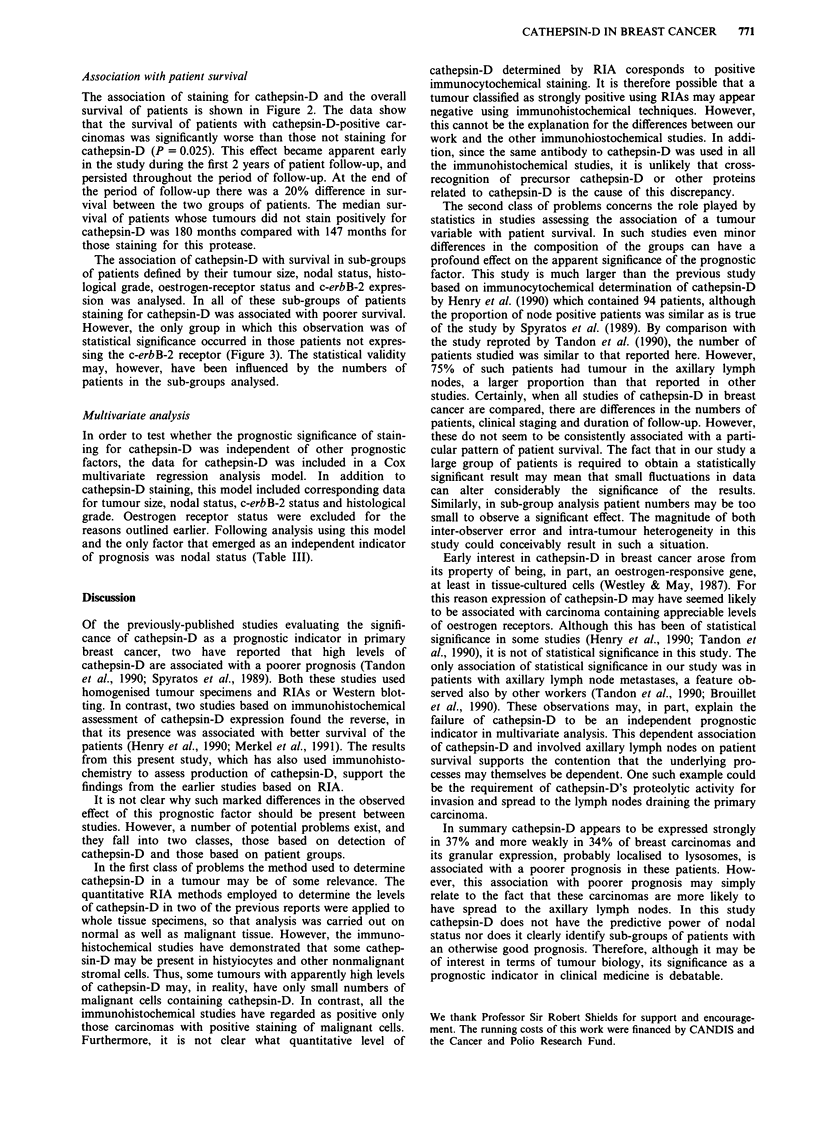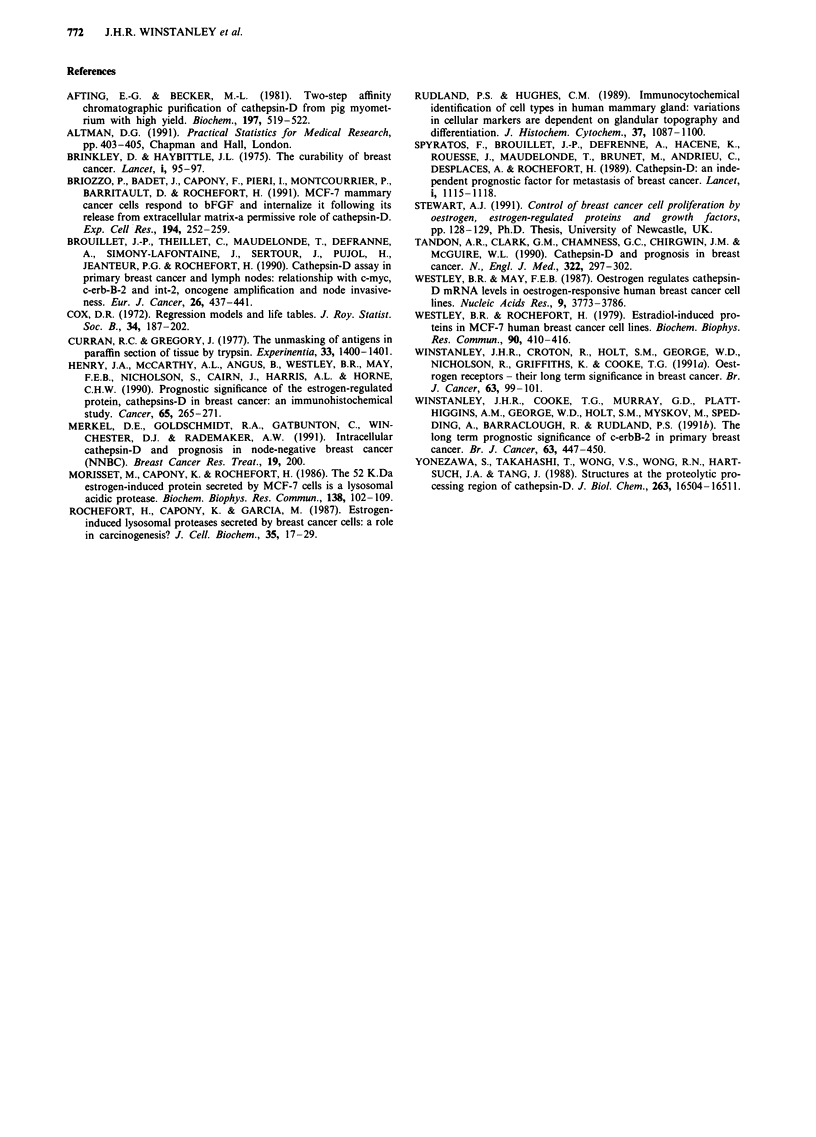# Prognostic significance of cathepsin-D in patients with breast cancer.

**DOI:** 10.1038/bjc.1993.139

**Published:** 1993-04

**Authors:** J. H. Winstanley, S. J. Leinster, T. G. Cooke, B. R. Westley, A. M. Platt-Higgins, P. S. Rudland

**Affiliations:** Department of Surgery, University of Liverpool Medical School, UK.

## Abstract

**Images:**


Br. J. Cancer (1993), 67, 767–772 © Macmillan Press Ltd., 1993

### Prognostic significance of cathepsin-D in patients with breast cancer

J.H.R. Winstanley[1], S.J. Leinster[1], T.G. Cooke[2], B.R. Westley[3], A.M. Platt-Higgins[4] & P.S. Rudland[4]

[1]*Department of Surgery, University of Liverpool Medical School, PO Box 147, Liverpool L69 3BX;* [2]*Department of Surgery, University of Glasgow Medical School, Glasgow;* [3]*Department of Pathology, University of Newcastle Medical School, Newcastle Upon Tyne NE1 4LP;* [4]*Cancer and Polio Research Fund Laboratories, Department of Biochemistry, University of Liverpool L69 3BX, UK.*

**Summary** The expression of the protease cathepsin-D has been evaluated using an immunohistochemical technique with a polyclonal antibody in paraffin-embedded tissue from 359 patients treated between the years 1975–1981 for Stage I and II breast cancer. One hundred and twenty seven patients (35%) have strongly positive, granular staining, 138 (38%) are intermediately stained in the cytoplasm, and in 94 (26%) no staining is observed. There is a strong positive association between expression of cathepsin-D and the presence of tumour in axillary lymph nodes ($P < 0.006$). Expression of the protease is associated with significantly poorer survival of patients in univariate analysis ($P = 0.025$); however, this is not independent of other tumour variables.

A significant feature of current research in breast cancer has been the contribution of biological factors in determining prognosis in groups of patients. Amongst the factors which have been the subject of such attention recently is the protease cathepsin-D (Spyratos *et al.*, 1989; Tandon *et al.*, 1990). This protease was originally identified as a protein secreted by oestrogen receptor-expressing cell lines after they were stimulated with oestrogen (Westley & Rochefort, 1979; Morisset *et al.*, 1986). Several forms of this protease exist, these include a precursor, procathepsin-D with a molecular weight of 52 kilodaltons (Kd). The procathepsin-D is digested proteolytically in lysosomes to a 48 Kd form and a more stable 34 Kd form (Yonezawa *et al.*, 1988). Biologically the molecule acts as a protease, active against basement membrane proteins and proteoglycans (Rochefort *et al.*, 1987). Some studies have suggested that it is mitogenic for oestrogen-depleted MCF-7 cells (Rochefort *et al.*, 1987); however, other workers have not been able to confirm this finding (Stewart, 1991). In addition, cathepsin-D secreted by MCF-7 cells participates in the mobilisation of extracellular matrix-bound basic fibroblast growth factor (Briozzo *et al.*, 1991).

In view of the above observations, a number of studies have been conducted on tumour homogenates from patients with breast cancer using a radioimmune assay (RIA) or Western blotting to determine whether cathepsin-D has any clinical relevance. These studies have suggested that a high cytosolic concentration of cathepsin-D is associated with a poor prognosis in primary breast cancer. Furthermore, the association of a high cytosolic concentration of cathepsin-D in patients with a poor prognosis may be independent of other prognostic factors, thus rendering it of particular value in identifying those patients with no detectable spread of the primary tumour to the axillary lymph nodes, but who have a poor prognosis (Spyratos *et al.*, 1989; Tandon *et al.*, 1990). However, studies in which the expression of cathepsin-D has been assessed in primary breast cancer using immunohistochemical analysis find ostensibly the reverse result, namely the presence of immunocytochemically detectable cathepsin-D is associated with a better prognosis in such patients (Henry *et al.*, 1990; Merkel *et al.*, 1991). These studies were not conducted on large numbers of patients and the period of follow-up was relatively short.

The disease of breast cancer has a long natural history (Brinkley & Haybittle, 1975) and it has been demonstrated that the duration of follow-up may influence the apparent significance of prognostic factors which are associated with the death of the patient (Winstanely *et al.*, 1991*a*). In order to address this problem with regards to cathepsin-D and clarify the apparently contradictory findings from earlier studies, we have evaluated the expression and clinical significance of cathepsin-D expression in primary breast carcinomas using immunohistochemical techniques for a large group of patients in whom long-term follow-up data is available (Winstanley *et al.*, 1991*b*).

### Materials and methods

### *Patients, specimens and serology*

Archival formalin-fixed, paraffin-embedded specimens were obtained for 359 patients, all of whom had presented with primary, operable breast cancer between the years 1976–1982 in the Merseyside region, as reported previously (Winstanley *et al.*, 1991*b*). Treatment was either modified radical mastectomy or simple mastectomy with sampling of axillary lymph nodes. The mean age of patients was 57 years (range 29–92). The distribution of the variables:tumour size, nodal status and histological grade is shown in Table I. All the patients had invasive carcinomas.

The rabbit polyclonal antiserum to cathepsin-D was raised against mature cathepsin-D which had been purified from human spleen by the method of Afting and Becker (1981). Polyacrylamide gel electrophoresis of the protein used for immunisation gave a single band of 34 Kd. The antiserum reacted predominantly with a protein of 34 Kd on Western blots of the total protein extracts of MCF-7 breast cancer cells, but minor bands of higher molecular weight (52 Kd, 48 Kd) corresponding to the precursor forms were also identified (Henry *et al.*, 1990).

### *Immunocytochemistry*

Histological sections were cut from the paraffin-embedded specimens and were incubated with the polyclonal antibody to cathepsin-D. The primary antibody was localised by the peroxidase-antiperoxidase (PAP) method, as described previously, including the blocking of endogenous peroxidase with methanol/0.05% $H_2O_2$ (v/v) and the non-specific serum-binding sites with 10% normal swine serum (Winstanley *et al.*, 1991*b*). However, after blocking the endogenous peroxidase activity, the histological sections were partially digested with 0.1% trypsin (w/v) in 0.1% $CaCl_2$ (w/v) for 10 min at 37°C (Curran & Gregory, 1977), as described for the use of the anti-cathepsin-D serum (Henry *et al.*, 1990). The primary

Correspondence: J.H.R. Winstanley.
Received 5 June 1992; and in revised form 8 October 1992.

**Table I** Distribution of tumour variables, tumour size, nodal status and histological grade between patients

| *Tumour variable*[a] | *Number of patients (%)* |
|---|---|
| $T_1$ | 30 (9%) |
| T2 | 241 (71%) |
| $T_3$ | 65 (20%) |
| N − | 229 (64%) |
| N + | 130 (36%) |
| G1 | 50 (27%) |
| G2 | 78 (43%) |
| G3 | 52 (30%) |

[a]Abbreviations: T, tumour size using the normal convention (Winstanley *et al.*, 1991*a*); N, involved (+) or uninvolved (−) lymph nodes; G, histological grade (Winstanley *et al.*, 1991*a*).

antibody was used at 1/250 for 90 min at room temperature and a red coloration due to bound peroxidase was developed with 3-amino-9-ethyl carbazole (Polyscience Ltd, Northampton, UK). The cellular nuclei were counterstained blue with Mayers haemalum.

Slides were read independently by two observers using light microscopy. Histological sections were regarded as positive if granular cytoplasmic staining were present in malignant cells, as reported previously (Henry *et al.*, 1990). Staining was evaluated initially in three groups: strong, intermediate, and absent, based on whether the staining occurred in cytoplasmic granules, throughout the cytoplasm but not concentrated in granules, or was absent altogether. A minimum of two sections and ten fields per section at 200 × magnification were analysed. Photographs were recorded on a Reichert Polyvar microscope fitted with a Wratten 44 blue-green filter (Rudland & Hughes, 1989).

Consistency of immunocytochemical staining between batches was checked by including in each batch of 20 sections, a standard histological section from a known positive specimen. Sections stained without anti-cathepsin-D serum or with the primary antibody preincubated with cathepsin-D showed no positive staining. Increasing the concentration of primary antibody 5-fold failed to stain any additional sections.

### *Statistical analysis*

Follow-up data had previously been obtained from the Merseyside Cancer Registry for the patients used in this study and this data for patient survival was updated in January 1990 (Winstanley *et al.*, 1991*b*). The association of cathepsin-D with other tumour variables was assessed using a simple Chi-square test (Altman, 1991). Since quantitation of oestrogen receptors was available for a number of the original tumours removed at the time of presentation, the association of cathepsin-D with both the presence and the level of oestrogen receptors was investigated. The presence of oestrogen receptors was set at a level above 5 fmol $mg^{-1}$ of cytosolic protein (Winstanley *et al.*, 1991*a*). The significance of the association between the levels of oestrogen receptor in fmol $mg^{-1}$ protein and cathepsin-D was evaluated by a Mann-Whitney U-test (Altman, 1991).

The association of the production of cathepsin-D in breast cancers with patient survival was evaluated using life tables constructed from survival data according to the method described by Kaplan and Meier, and analysed using a log-rank sum (Winstanley *et al.*, 1991*b*). In order to determine whether the association of patient survival with cathepsin-D was independent of other prognostic factors shown to be significant in univariate analysis, a multivariate analysis of these factors was carried out using a Cox multiple regression model (Cox, 1972). Other prognostic factors measured on the same group of patients included tumour size, nodal status and the presence of the oncogene c-*erb*B-2 (Winstanley *et al.*, 1991*b*).

Histological grade was included in this analysis, but data relating to this prognostic factor was available only for a smaller sub-set of the whole group of patients. Since the presence of oestrogen receptors has been shown previously to lack statistical significance in our group of patients, this factor was also excluded from the Cox multiple regression analysis (Winstanley *et al.*, 1991*a*). The degree of correlation between observers was assessed using the Kappa statistic, a value of greater than 0.61 was taken to represent a satisfactory level of agreement (Altman, 1991).

### Results

Of the 359 breast carcinomas evaluated, 127 (35%) of them had strong granular, cytoplasmic staining (Figure 1a,b), 138 (38%) were intermediately stained in their cytoplasm (Figure 1c) and the remaining 94 (26%) did not stain (Figure 1d). The assessment was made only on the malignant cells; however, positive staining was also present in normal histiocytes, macrophages and blood vessel walls (Figure 1e). Sometimes atypical elements of benign breast proliferations within the primary breast carcinoma were also stained for cathepsin-D, but they were always associated with staining of the carcinoma cells (Figure 1f). The mean period of follow-up of the patients was 11 years (range 8–16 years), and their average age was 57 years at the time of presentation. For the purposes of analysis both strongly stained and intermediately stained carcinomas were combined into one group of positively stained tumours.

### *Interobserver and intratumour variation*

There was some variability in the assessment of staining by the two observers on the same histological section. However, there was agreement in 90% of the slides; this corresponded to a Kappa value of 0.85, which represents a high degree of consistency between observers. In 4% of all the slides studied, intratumour heterogeneity affected these slides were regarded as negatively or positively stained. Intratumour heterogeneity was assessed by comparing the type of staining allocated when two sections from the same tumour were read independently.

### *Association with other tumour variables*

The presence of definite staining for cathepsin-D was cross-tabulated with other tumour variables using a simple Chi-square test (Table II). These included tumour size, nodal

**Table II** Association of cathepsin-D expression with other tumour variables

| *Tumour variable*[a] | *Cathepsin-D-negative*[b], *no (%)* | *Cathepsin-D-positive*[b], *no (%)* | *Statistical significance*[c] |
|---|---|---|---|
| T1 | 7 (8) | 23 (9) | |
| T2 | 59 (71) | 182 (71) | $P = 0.95$ |
| T3 | 17 (21) | 48 (19) | |
| N − | 71 (75) | 158 (59) | $P = 0.006$ |
| N + | 23 (25) | 107 (41) | |
| G1 | 17 (37) | 33 (24) | |
| G2 | 21 (45) | 57 (43) | $P = 0.09$ |
| G3 | 8 (18) | 44 (33) | |
| Erb + | 20 (26) | 73 (26) | $P = 0.2$ |
| Erb − | 73 (74) | 202 (74) | |
| ER + | 50 (54) | 154 (59) | $P = 0.4$ |
| ER − | 42 (46) | 105 (41) | |

[a]Abbreviations: T, tumour size using the normal convention (Winstanley *et al.*, 1991*a*); N, involved (+) or uninvolved (−) lymph nodes; G, histological grade (Winstanley *et al.*, 1991*a*); Erb, the presence (+) or absence (−) of the c-*erb*B-2 receptor (Winstanley *et al.*, 1991*b*); and ER, the presence (+) or absence (−) of the oestrogen receptor (Winstanley *et al.*, 1991*a*). [b]Number of patients with tumours either staining (+) or not staining (−) for cathepsin-D. Parentheses contain the percentage of patients. [c]Probability, *P* from Chi-square test.

**Figure 1** Immunocytochemical staining of tumours using anti-cathepsin-D. **a**, Invasive carcinoma showing strong positive staining for anti-cathepsin-D. Bar = 50 µm, × 220. **b**, Higher magnification of A showing the granular nature of the cytoplasmic staining. Bar = 20 µm, × 550. **c**, Weak, speckled staining for anti-cathepsin-D of invasive carcinoma (arrows) with strong staining of host cells (arrowheads). Bar = 50 µm, × 220. **d**, invasive carcinoma showing no staining for cathepsin-D. Bar = 50 µm, × 220. **e**, Host blood vessels stained positively for cathepsin-D. Bar = 50 µm, × 220. **f**, Area of epithelial hyperplasia in an invasive carcinoma showing positive staining of atypical elements. Bar = 50 µm, × 180.

status, histological grade, oestrogen receptor status and the presence of c-*erb*B-2. The strongest statistical association was observed between the presence of staining for cathepsin-D and the involvement of axillary nodes with tumour; 38% of cathepsin-D positive tumours had tumour in the associated axillary lymph nodes compared with only 25% of those that were cathepsin-D negative ($P = 0.006$). There was a tendency for more oestrogen-receptor positive carcinomas to be positive also for cathepsin-D, when compared with the oestrogen-receptor negative carcinomas. However, this tendency did not achieve statistical significance ($P = 0.4$). Similarly, the presence of cathepsin-D staining was associated with poorly differentiated tumours; but, as in the case of oestrogen receptors, this association did not achieve statistical significance ($P = 0.09$). The presence of cathepsin-D was also not associated in any significance with higher levels of oestrogen receptors using the Mann-Whitney U-test ($P = 0.5$).

**Table III** Dependence of the prognostic significance of staining for cathepsin-D on other prognostic factors using Cox multivariate regression analysis

| Prognostic factor[a] | Cox multivariate regression analysis[b] Coefficient (β) | Standard error (SE) | Coefficient ÷ SE (Z-value)[c] |
|---|---|---|---|
| N | 0.77 | 0.20 | 3.85 |
| Erb | 0.135 | 0.30 | 0.67 |
| $T_2$ | −0.28 | 0.31 | −0.90 |
| $T_3$ | 0.136 | 0.34 | 0.40 |
| Cathepsin | 0.068 | 0.12 | 0.56 |
| Grade | 0.114 | 0.08 | 1.42 |

[a]Abbreviations are as in Table I; N for involved lymph nodes; Erb, presence of c-*erb*B-2 receptor; $T_2$ and $T_3$, tumour size; cathepsin, presence of cathepsin-D, and grade, histological grade. [b]The values of parameters of the Cox multivariate regression analysis are shown. [c]Overall Chi-square = 22.65 for 5 degrees of freedom; $P = 0.0004$.

**Figure 2** Association of staining for cathepsin-D with overall survival of the patients. The cumulative proportion of surviving patients as a percentage of the total is shown for each year after presentation for either **a**, patients with cathepsin-D-negatively staining (——) or **b**, cathepsin-D-positively-staining carcinomas (---). 100% for cathepsin-D-negative carcinomas corresponds to 94 patients, and 100% for cathepsin-D-positive carcinomas corresponds to 265 patients. The two curves are significantly different (Chi-square = 4.98, 1 degree of freedom, $P = 0.025$).

**Figure 3** Association of staining for cathepsin-D with survival of patients divided into groups by their c-*erb*B-2 status. The cumulative proportion of surviving patients as a percentage of the total is shown for each year after presentation for the following: **a**, patients with cathepsin-D-negative, c-*erb*B-2-negative carcinomas (——) (100% = 71 patients); **b**, patients with cathepsin-D-positive, c-*erb*B-2-negative carcinoma (----) (100% = 199 patients); **c**, patients with cathepsin-D-positive, c-*erb*B-2-positive carcinomas (----) (100% = 56 patients); **d**, patients with cathepsin-D-negative, c-*erb*B-2-positive carcinomas (——) (100% = 20 patients). The cathepsin-D-negative, c-*erb*B-2-negative **a**, and cathepsin-D-positive, c-*erb*B-2-negative **b**, curves are significantly different (Chi-square = 9.2, 1 degree of freedom, $P = 0.0024$); but the cathepsin-D-positive, c-*erb*B-2-positive **c**, and cathepsin-D-negative, c-*erb*B-2-positive **d**, curves are not significantly different (Chi-square = 0.27, 1 degree of freedom, $P = 0.6$). Data for c-*erb*B-2 status is available only for 346 out of the 359 patients.

*Association with patient survival*

The association of staining for cathepsin-D and the overall survival of patients is shown in Figure 2. The data show that the survival of patients with cathepsin-D-positive carcinomas was significantly worse than those not staining for cathepsin-D ($P = 0.025$). This effect became apparent early in the study during the first 2 years of patient follow-up, and persisted throughout the period of follow-up. At the end of the period of follow-up there was a 20% difference in survival between the two groups of patients. The median survival of patients whose tumours did not stain positively for cathepsin-D was 180 months compared with 147 months for those staining for this protease.

The association of cathepsin-D with survival in sub-groups of patients defined by their tumour size, nodal status, histological grade, oestrogen-receptor status and c-*erb*B-2 expression was analysed. In all of these sub-groups of patients staining for cathepsin-D was associated with poorer survival. However, the only group in which this observation was of statistical significance occurred in those patients not expressing the c-*erb*B-2 receptor (Figure 3). The statistical validity may, however, have been influenced by the numbers of patients in the sub-groups analysed.

*Multivariate analysis*

In order to test whether the prognostic significance of staining for cathepsin-D was independent of other prognostic factors, the data for cathepsin-D was included in a Cox multivariate regression analysis model. In addition to cathepsin-D staining, this model included corresponding data for tumour size, nodal status, c-*erb*B-2 status and histological grade. Oestrogen receptor status were excluded for the reasons outlined earlier. Following analysis using this model and the only factor that emerged as an independent indicator of prognosis was nodal status (Table III).

**Discussion**

Of the previously-published studies evaluating the significance of cathepsin-D as a prognostic indicator in primary breast cancer, two have reported that high levels of cathepsin-D are associated with a poorer prognosis (Tandon *et al.*, 1990; Spyratos *et al.*, 1989). Both these studies used homogenised tumour specimens and RIAs or Western blotting. In contrast, two studies based on immunohistochemical assessment of cathepsin-D expression found the reverse, in that its presence was associated with better survival of the patients (Henry *et al.*, 1990; Merkel *et al.*, 1991). The results from this present study, which has also used immunohistochemistry to assess production of cathepsin-D, support the findings from the earlier studies based on RIA.

It is not clear why such marked differences in the observed effect of this prognostic factor should be present between studies. However, a number of potential problems exist, and they fall into two classes, those based on detection of cathepsin-D and those based on patient groups.

In the first class of problems the method used to determine cathepsin-D in a tumour may be of some relevance. The quantitative RIA methods employed to determine the levels of cathepsin-D in two of the previous reports were applied to whole tissue specimens, so that analysis was carried out on normal as well as malignant tissue. However, the immunohistochemical studies have demonstrated that some cathepsin-D may be present in histyiocytes and other nonmalignant stromal cells. Thus, some tumours with apparently high levels of cathepsin-D may, in reality, have only small numbers of malignant cells containing cathepsin-D. In contrast, all the immunohistochemical studies have regarded as positive only those carcinomas with positive staining of malignant cells. Furthermore, it is not clear what quantitative level of cathepsin-D determined by RIA coresponds to positive immunocytochemical staining. It is therefore possible that a tumour classified as strongly positive using RIAs may appear negative using immunohistochemical techniques. However, this cannot be the explanation for the differences between our work and the other immunohiostochemical studies. In addition, since the same antibody to cathepsin-D was used in all the immunohistochemical studies, it is unlikely that cross-recognition of precursor cathepsin-D or other proteins related to cathepsin-D is the cause of this discrepancy.

The second class of problems concerns the role played by statistics in studies assessing the association of a tumour variable with patient survival. In such studies even minor differences in the composition of the groups can have a profound effect on the apparent significance of the prognostic factor. This study is much larger than the previous study based on immunocytochemical determination of cathepsin-D by Henry *et al.* (1990) which contained 94 patients, although the proportion of node positive patients was similar as is true of the study by Spyratos *et al.* (1989). By comparison with the study reproted by Tandon *et al.* (1990), the number of patients studied was similar to that reported here. However, 75% of such patients had tumour in the axillary lymph nodes, a larger proportion than that reported in other studies. Certainly, when all studies of cathepsin-D in breast cancer are compared, there are differences in the numbers of patients, clinical staging and duration of follow-up. However, these do not seem to be consistently associated with a particular pattern of patient survival. The fact that in our study a large group of patients is required to obtain a statistically significant result may mean that small fluctuations in data can alter considerably the significance of the results. Similarly, in sub-group analysis patient numbers may be too small to observe a significant effect. The magnitude of both inter-observer error and intra-tumour heterogeneity in this study could conceivably result in such a situation.

Early interest in cathepsin-D in breast cancer arose from its property of being, in part, an oestrogen-responsive gene, at least in tissue-cultured cells (Westley & May, 1987). For this reason expression of cathepsin-D may have seemed likely to be associated with carcinoma containing appreciable levels of oestrogen receptors. Although this has been of statistical significance in some studies (Henry *et al.*, 1990; Tandon *et al.*, 1990), it is not of statistical significance in this study. The only association of statistical significance in our study was in patients with axillary lymph node metastases, a feature observed also by other workers (Tandon *et al.*, 1990; Brouillet *et al.*, 1990). These observations may, in part, explain the failure of cathepsin-D to be an independent prognostic indicator in multivariate analysis. This dependent association of cathepsin-D and involved axillary lymph nodes on patient survival supports the contention that the underlying processes may themselves be dependent. One such example could be the requirement of cathepsin-D's proteolytic activity for invasion and spread to the lymph nodes draining the primary carcinoma.

In summary cathepsin-D appears to be expressed strongly in 37% and more weakly in 34% of breast carcinomas and its granular expression, probably localised to lysosomes, is associated with a poorer prognosis in these patients. However, this association with poorer prognosis may simply relate to the fact that these carcinomas are more likely to have spread to the axillary lymph nodes. In this study cathepsin-D does not have the predictive power of nodal status nor does it clearly identify sub-groups of patients with an otherwise good prognosis. Therefore, although it may be of interest in terms of tumour biology, its significance as a prognostic indicator in clinical medicine is debatable.

We thank Professor Sir Robert Shields for support and encouragement. The running costs of this work were financed by CANDIS and the Cancer and Polio Research Fund.

### References

AFTING, E.-G. & BECKER, M.-L. (1981). Two-step affinity chromatographic purification of cathepsin-D from pig myometrium with high yield. *Biochem.*, **197**, 519–522.

ALTMAN, D.G. (1991). *Practical Statistics for Medical Research*, pp. 403–405, Chapman and Hall, London.

BRINKLEY, D. & HAYBITTLE, J.L. (1975). The curability of breast cancer. *Lancet*, **i**, 95–97.

BRIOZZO, P., BADET, J., CAPONY, F., PIERI, I., MONTCOURRIER, P., BARRITAULT, D. & ROCHEFORT, H. (1991). MCF-7 mammary cancer cells respond to bFGF and internalize it following its release from extracellular matrix-a permissive role of cathepsin-D. *Exp. Cell Res.*, **194**, 252–259.

BROUILLET, J.-P., THEILLET, C., MAUDELONDE, T., DEFRANNE, A., SIMONY-LAFONTAINE, J., SERTOUR, J., PUJOL, H., JEANTEUR, P.G. & ROCHEFORT, H. (1990). Cathepsin-D assay in primary breast cancer and lymph nodes: relationship with c-myc, c-erb-B-2 and int-2, oncogene amplification and node invasiveness. *Eur. J. Cancer*, **26**, 437–441.

COX, D.R. (1972). Regression models and life tables. *J. Roy. Statist. Soc. B.*, **34**, 187–202.

CURRAN, R.C. & GREGORY, J. (1977). The unmasking of antigens in paraffin section of tissue by trypsin. *Experinentia*, **33**, 1400–1401.

HENRY, J.A., MCCARTHY, A.L., ANGUS, B., WESTLEY, B.R., MAY, F.E.B., NICHOLSON, S., CAIRN, J., HARRIS, A.L. & HORNE, C.H.W. (1990). Prognostic significance of the estrogen-regulated protein, cathepsins-D in breast cancer: an immunohistochemical study. *Cancer*, **65**, 265–271.

MERKEL, D.E., GOLDSCHMIDT, R.A., GATBUNTON, C., WINCHESTER, D.J. & RADEMAKER, A.W. (1991). Intracellular cathepsin-D and prognosis in node-negative breast cancer (NNBC). *Breast Cancer Res. Treat.*, **19**, 200.

MORISSET, M., CAPONY, K. & ROCHEFORT, H. (1986). The 52 K.Da estrogen-induced protein secreted by MCF-7 cells is a lysosomal acidic protease. *Biochem. Biophys. Res. Commun.*, **138**, 102–109.

ROCHEFORT, H., CAPONY, K. & GARCIA, M. (1987). Estrogen-induced lysosomal proteases secreted by breast cancer cells: a role in carcinogenesis? *J. Cell. Biochem.*, **35**, 17–29.

RUDLAND, P.S. & HUGHES, C.M. (1989). Immunocytochemical identification of cell types in human mammary gland: variations in cellular markers are dependent on glandular topography and differentiation. *J. Histochem. Cytochem.*, **37**, 1087–1100.

SPYRATOS, F., BROUILLET, J.-P., DEFRENNE, A., HACENE, K., ROUESSE, J., MAUDELONDE, T., BRUNET, M., ANDRIEU, C., DESPLACES, A. & ROCHEFORT, H. (1989). Cathepsin-D: an independent prognostic factor for metastasis of breast cancer. *Lancet*, **i**, 1115–1118.

STEWART, A.J. (1991). *Control of breast cancer cell proliferation by oestrogen, estrogen-regulated proteins and growth factors*, pp. 128–129, Ph.D. Thesis, University of Newcastle, UK.

TANDON, A.R., CLARK, G.M., CHAMNESS, G.C., CHIRGWIN, J.M. & MCGUIRE, W.L. (1990). Cathepsin-D and prognosis in breast cancer. *N., Engl. J. Med.*, **322**, 297–302.

WESTLEY, B.R. & MAY, F.E.B. (1987). Oestrogen regulates cathepsin-D mRNA levels in oestrogen-responsive human breast cancer cell lines. *Nucleic Acids Res.*, **9**, 3773–3786.

WESTLEY, B.R. & ROCHEFORT, H. (1979). Estradiol-induced proteins in MCF-7 human breast cancer cell lines. *Biochem. Biophys. Res. Commun.*, **90**, 410–416.

WINSTANLEY, J.H.R., CROTON, R., HOLT, S.M., GEORGE, W.D., NICHOLSON, R., GRIFFITHS, K. & COOKE, T.G. (1991*a*). Oestrogen receptors – their long term significance in breast cancer. *Br. J. Cancer*, **63**, 99–101.

WINSTANLEY, J.H.R., COOKE, T.G., MURRAY, G.D., PLATT-HIGGINS, A.M., GEORGE, W.D., HOLT, S.M., MYSKOV, M., SPEDDING, A., BARRACLOUGH, R. & RUDLAND, P.S. (1991*b*). The long term prognostic significance of c-erbB-2 in primary breast cancer. *Br. J. Cancer*, **63**, 447–450.

YONEZAWA, S., TAKAHASHI, T., WONG, V.S., WONG, R.N., HARTSUCH, J.A. & TANG, J. (1988). Structures at the proteolytic processing region of cathepsin-D. *J. Biol. Chem.*, **263**, 16504–16511.